# Man with Penile Pain

**DOI:** 10.5811/cpcem.2020.7.47903

**Published:** 2020-07-20

**Authors:** Jesse Wray, Rachel E. Bridwell, Michael J. Yoo, Christopher N. Belcher, Joshua J. Oliver

**Affiliations:** San Antonio Uniformed Services Health Education Consortium, Department of Emergency Medicine, Fort Sam Houston, Texas

**Keywords:** Thrombosis of corpus cavernosum, partial priapism, penile thrombus

## Abstract

**Case Presentation:**

We describe a case of spontaneous partial segmental thrombosis of the corpus cavernosum (PSTCC).

**Discussion:**

PSTCC is a rare condition in which thrombus formation occurs in the corpus cavernosum. This condition is managed in conjunction with a urologist, and management typically includes anticoagulation and pain control.

## CASE PRESENTATION

A 39-year-old man presented to the emergency department for two days of worsening pain and swelling to the base of his penis. The patient denied trauma or a history of coagulopathy, had a non-contributory sexual history, and no recent use of erectile dysfunction medications. Examination demonstrated mild swelling to the penile base without evidence of hernia, infection, or shaft injury.

**Image 1:**
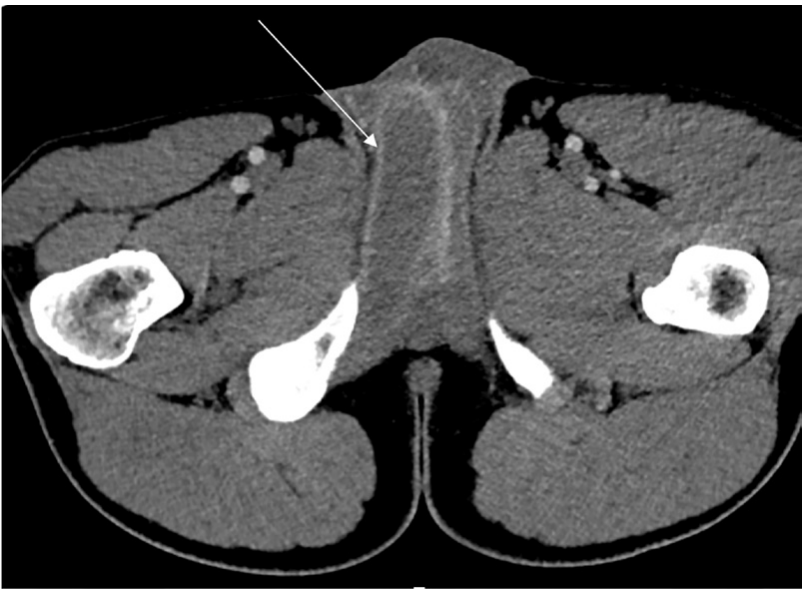
Non-contrasted computed tomography of the pelvis demonstrating asymmetry of the right and left corpus cavernosum.

**Image 2:**
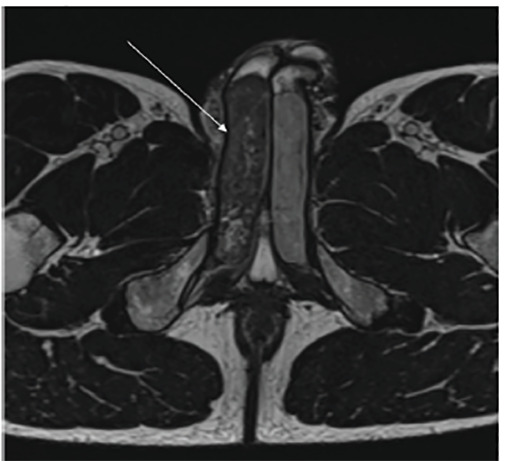
Axial T2-weighted magnetic resonance imaging of the pelvis revealing a hypointense signal of the right corpus cavernosum.

**Image 3:**
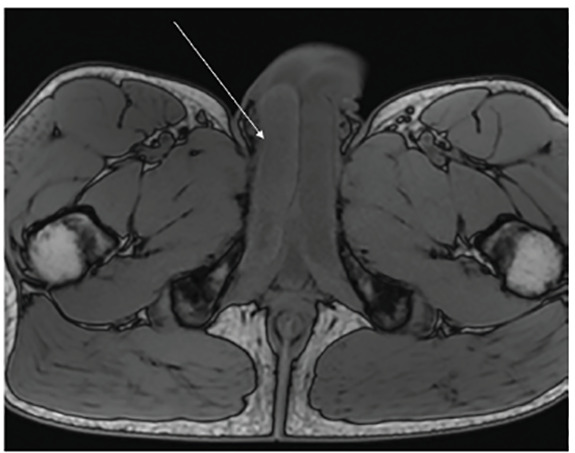
Axial T1-weighted magnetic resonance imaging of the pelvis with hyperintense signal of the right corpus cavernosum.

Computed tomography (CT) revealed penile asymmetry (Image 1). Ultrasound demonstrated asymmetric fullness of the right corpus cavernosum. Pelvis magnetic resonance imaging (MRI) revealed an enlarged appearance of the right corpus cavernosum with hypointense T2 signal (Image 2) and hyperintense T1 signal (Image 3). These findings were consistent with a partial segmental thrombosis of the right corpus cavernosum (PSTCC). The patient was admitted for pain control and discharged after symptom resolution with anticoagulation therapy. Upon outpatient follow-up, the patient had no persistent complications.

## DISCUSSION

PSTCC is a rare condition that manifests as penile or perineal pain and swelling. Thrombus formation likely arises secondary to microtrauma, thrombophilia, hemoglobinopathies and, rarely, medication side effect.^1^,^2^ Ultrasonography or MRI are recommended diagnostic modalities, while CT is reportedly suboptimal due to decreased sensitivity for this condition.^3^ Our case departs from the literature as CT and MRI were most useful. Additionally, because CT clearly demonstrates the pathology in this case, it may be a better diagnostic modality than previously reported in this rare phenomenon and serve as a rapid diagnostic tool in some cases. Early urologic consultation is recommended, with typical management consisting of anticoagulation and pain control.^2^ PSTCC has an overall favorable prognosis rarely incurring long-term complications.^3^

**CPC-EM Capsule**

What do we already know about this clinical entity?

Partial segmental thrombosis of the corpus cavernosum (PSTCC) is a rare condition classically diagnosed with ultrasound or magnetic resonance imaging.

What is the major impact of the image(s)?

Although computed tomography (CT) has not been previously recommended for identifying this pathology, our case demonstrates that PSTCC can be clearly identified with CT.

How might this improve emergency medicine practice?

This example of a rare pathology that may go unrecognized by emergency providers demonstrates the use of CT to aid in diagnosis.
